# Dark blood cardiovascular magnetic resonance of the heart, great vessels, and lungs using electrocardiographic-gated three-dimensional unbalanced steady-state free precession

**DOI:** 10.1186/s12968-021-00808-2

**Published:** 2021-11-01

**Authors:** Robert R. Edelman, Nondas Leloudas, Jianing Pang, Ioannis Koktzoglou

**Affiliations:** 1 Department of Radiology, Northshore University HealthSystem, Evanston, IL USA; 2 Department of Radiology, Feinberg School of Medicine, Northwestern University, Chicago, IL USA; 3 Siemens Medical Solutions USA Inc., Chicago, IL USA; 4 Radiology, Pritzker School of Medicine, University of Chicago, Chicago, IL USA; 5 Walgreen Building, G534, 2650 Ridge Avenue, Evanston, IL 60201 USA

**Keywords:** T1 relaxation-enhanced steady-state, Unbalanced 3D steady-state free precession, Breath-hold, Navigator gating, Electrocardiographic (ECG) gating, Magnetic resonance, Dark blood imaging, Cardiac, Great vessels, Lungs

## Abstract

**Background:**

Recently, we reported a novel neuroimaging technique, unbalanced T1 Relaxation-Enhanced Steady-State (uT_1_RESS), which uses a tailored 3D unbalanced steady-state free precession (3D uSSFP) acquisition to suppress the blood pool signal while minimizing bulk motion sensitivity. In the present work, we hypothesized that 3D uSSFP might also be useful for dark blood imaging of the chest. To test the feasibility of this approach, we performed a pilot study in healthy subjects and patients undergoing cardiovascular magnetic resonance (CMR).

**Main body:**

The study was approved by the hospital institutional review board. Thirty-one adult subjects were imaged at 1.5 T, including 5 healthy adult subjects and 26 patients (44 to 86 years, 10 female) undergoing a clinically indicated CMR. Breath-holding was used in 29 subjects and navigator gating in 2 subjects. For breath-hold acquisitions, the 3D uSSFP pulse sequence used a high sampling bandwidth, asymmetric readout, and single-shot along the phase-encoding direction, while 3 shots were acquired for navigator-gated scans. To minimize signal dephasing from bulk motion, electrocardiographic (ECG) gating was used to synchronize the data acquisition to the diastolic phase of the cardiac cycle. To further reduce motion sensitivity, the moment of the dephasing gradient was set to one-fifth of the moment of the readout gradient. Image quality using 3D uSSFP was good-to-excellent in all subjects. The blood pool signal in the thoracic aorta was uniformly suppressed with sharp delineation of the aortic wall including two cases of ascending aortic aneurysm and two cases of aortic dissection. Compared with variable flip angle 3D turbo spin-echo, 3D uSSFP showed improved aortic wall sharpness. It was also more efficient, permitting the acquisition of 24 slices in each breath-hold versus 16 slices with 3D turbo spin-echo and a single slice with dual inversion 2D turbo spin-echo. In addition, lung and mediastinal lesions appeared highly conspicuous compared with the low blood pool signals within the heart and blood vessels. In two subjects, navigator-gated 3D uSSFP provided excellent delineation of cardiac morphology in double oblique multiplanar reformations.

**Conclusion:**

In this pilot study, we have demonstrated the feasibility of using ECG-gated 3D uSSFP for dark blood imaging of the heart, great vessels, and lungs. Further study will be required to fully optimize the technique and to assess clinical utility.

## Background

Cardiovascular magnetic resonance (CMR) imaging techniques can be broadly classified as bright blood, gray blood, or dark blood. Three-dimensional (3D) bright blood techniques are used to create CMR angiograms by depicting the arterial lumen [1], whereas 3D gray blood [2] and dark blood [3] techniques are primarily used to evaluate the arterial wall [4]. Examples of 3D dark blood techniques include variable flip angle turbo spin-echo (VFA-TSE, also called SPACE, CUBE, or VISTA) [5], diffusion-prepared balanced steady-state free precession (bSSFP) [6], motion-sensitized driven equilibrium-prepared gradient-echo [7], delay alternating with nutation for tailored excitation (DANTE)-prepared gradient-echo [8], and simultaneous non-contrast angiography and intraplaque hemorrhage (SNAP) [9]. These techniques typically have scan times on the order of several minutes or longer and work most reliably in stationary regions such as the head and neck [10]. To apply these techniques in regions affected by respiratory motion, free-breathing acquisitions are used since scan times are too long to permit breath-holding [11–13].

Recently, we reported a novel prototype 3D dark blood neuroimaging technique, called unbalanced T1 Relaxation-Enhanced Steady-State (uT_1_RESS) [14]. uT_1_RESS substantially improves the visibility of tumors in contrast-enhanced MR by differentially suppressing the signal intensity of non-enhancing background tissues and blood vessels while maintaining the signal intensity of contrast-enhancing lesions. The uT_1_RESS technique uses a tailored 3D unbalanced steady-state free precession (3D uSSFP) readout to suppress the blood pool signals while minimizing bulk motion artifacts. Given the excellent image quality and uniform suppression of intravascular signals that has been obtained in the brain, we hypothesized that the tailored 3D uSSFP readout might also be useful for dark blood imaging of the cardiovascular system and lungs. Moreover, its high scan efficiency permits breath-hold imaging, which makes it an attractive technique for regions like the chest and abdomen that are affected by respiratory motion. To test the feasibility of this approach, we performed a pilot study of 3D uSSFP in healthy subjects and patients undergoing CMR, with particular attention to the heart, great vessels, and lungs.

## Methods

### Study cohort

The study was approved by the hospital institutional review board. Waiver of consent was obtained for patients undergoing a clinically indicated CMR during which additional dark blood sequences were obtained. Imaging was performed on a 1.5 T CMR system (MAGNETOM Avanto Dot, Aera, or Sola, Siemens Healthineers, Erlangen, Germany). 31 adult subjects were studied including 5 healthy adult subjects and 26 patients (44 to 86 years, 10 female). 6 subjects were scanned on the Aera, 1 on the Sola, and the remainder on the Avanto Dot. Breath-holding was used in 29 subjects and navigator gating in 2 subjects. Four patients had abnormalities of the thoracic aorta (aortic aneurysm, n = 2; aortic dissection, n = 2). Four patients had abnormalities of the lungs and/or mediastinum (benign lung nodule, n = 1; lung cancer, n = 1; sarcoid, n = 1; mediastinal cyst, n = 1) that were incidental to the clinical indication for the CMR exam. In 2 patients, 3D uSSFP was acquired after the administration of 0.2 mmol/kg of gadobutrol (Bayer Healthcare, Berlin, Germany). In the remainder, all dark blood sequences were acquired prior to contrast administration.

### 3D uSSFP pulse sequence

**Fig. 1:**
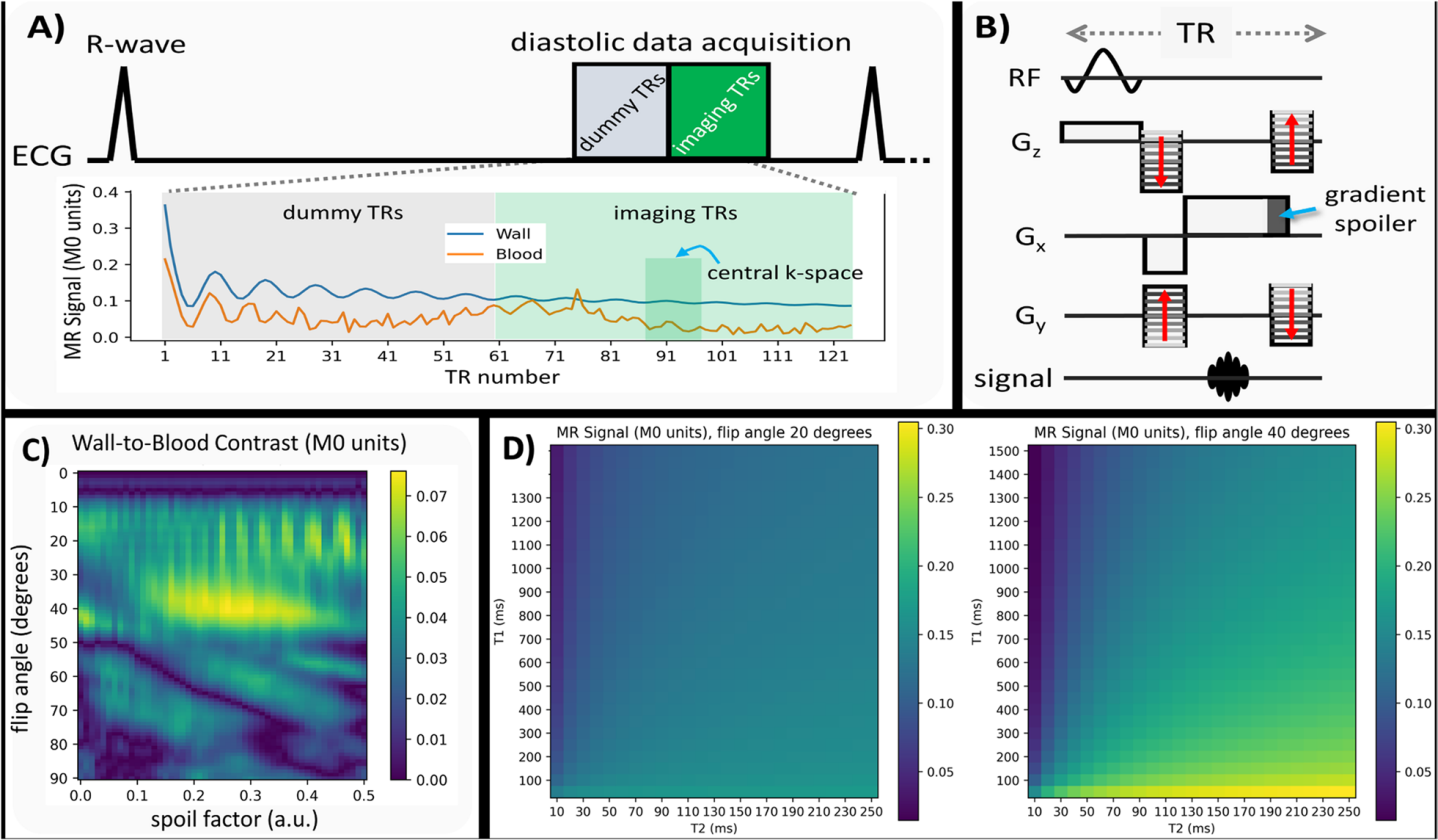
3D unbalanced T1 relaxation-enhances steady state free precession (uSSFP) pulse sequence. **A** Pulse sequence diagram showing the simulated signal evolution for the aortic wall and blood at a flip angle of 40° and spoiler factor of 0.2. The orange and blue lines correspond to arterial blood (T1 = 1200 ms, T2 = 250 ms, velocity = 15 cm/s) and arterial wall (T1 = 1000 ms, T2 = 50 ms, velocity = 0 cm/s), respectively. **B** uSSFP pulse sequence diagram spanning one TR showing gradient activity. **C** Plot of simulated aortic wall-to-blood signal contrast obtained with uSSFP over a range of acquisition flip angles and gradient spoiler factors for the central k-space region indicated in **A**. A flip angle of approximately 40° and a gradient spoiler factor of approximately 0.2 to 0.3 optimized arterial-to-blood contrast. Values are in equilibrium magnetization (M0) units. **D** Plot of mean uSSFP CMR signal for stationary tissue at the central 10 k-space lines as a function of tissue T1 and T2 for flip angles of 20° (left) and 40° (right)

The pulse sequence diagram for the uT_1_RESS-based 3D uSSFP technique is shown in Fig. 1A and B. To minimize bulk motion-related dephasing, electrocardiographic (ECG) gating was used to synchronize the data acquisition to the diastolic phase of the cardiac cycle. To further reduce motion sensitivity, the moment of the dephasing gradient was set to one-fifth of the moment of the readout gradient (gradient spoiler factor = 0.2). A series of constant-flip-angle dummy radiofrequency (RF) repetitions was applied immediately prior to each diastolic-gated shot to drive both inflowing and in-slab spins into the steady-state.

### Bloch equation modeling

Numerical simulations of the uSSFP sequence were performed to probe aortic wall-to-blood pool contrast and to determine mean signal for stationary tissue as a function of tissue T1 and T2. Simulations, which leveraged the methodology of extended phase graphs, were performed within the Sycomore MRI simulation toolkit (version 1.3.0, https://github.com/lamyj/sycomore/releases/tag/v1.3.0). Simulation parameters were as follows: T1/T2 for aortic wall and aortic blood were 1200 ms/250 ms and 1000 ms/50 ms, respectively; repetition time (TR) = 1.8 ms, echo time (TE) = 0.77 ms, voxel size = 2.6 mm, end-diastolic aortic blood pool and wall velocities of 15 cm/s and 0 cm/s [15], 60 dummy RF repetitions before each shot, 64 lines of k-space acquired during each shot, RR interval = 800 ms. Imaging RF flip angles ranging from 1 to 90° and gradient spoiler factors in the frequency-encoding direction ranging from 0.0 to 0.5 (corresponding to an additional 0 to π radians of dephasing per voxel) were simulated. Aortic wall-to-blood pool contrast was computed as the mean difference between aortic wall and aortic blood signal over the central 10 lines of k-space at the 4th simulated cardiac cycle. The mean uSSFP CMR signal for stationary tissue at the central 10 k-space lines was calculated as a function of tissue T1 and T2 for flip angles of 20° and 40°.

### Acquisition techniques

Breath-hold 3D uSSFP was acquired using a single shot along the phase-encoding direction, whereas navigator-gated 3D uSSFP used 3 shots. Pulse sequence parameters were selected based upon Bloch equation simulations and through empirical testing. Typical breath-hold sequence parameters included: 24 3D partitions acquired (reconstructed to 48 partitions) with 16.7% slice oversampling and 6/8 partial Fourier along the 3D partition direction; acquired 3D partition thickness = 2.6-mm with reconstructed thickness = 1.3-mm and in-plane resolution = 1.25-mm × 1.25-mm; field of view = 400-mm × 320-mm for oblique sagittal or axial imaging of the great vessels and 400-mm × 400-mm for coronal or axial imaging of the lungs; GRAPPA acceleration factor = 2 with 64 reference lines; 60 dummy pulses; sampling bandwidth = 1838 Hz/px; shot duration = 255 ms; echo spacing = 1.8 ms; TE = 0.77 ms using an asymmetric readout.

Navigator-gated 3D uSSFP used cross-pair navigators placed over the right diaphragm with an acceptance window of ± 2.5 mm and correction factor of 0.6. The shot duration was 144 ms with 36 dummy pulses. 48 to 64 3D partitions were acquired with reconstructed partition thickness of either 0.9-mm or 1.5-mm with in-plane resolution of 0.94-mm × 0.94-mm.

3D uSSFP was compared to standard 2D and 3D dark blood sequences (dual inversion 2D turbo spin-echo (DIR-TSE) [16] and 3D VFA-TSE) in 8 subjects (one healthy subject and 7 patients). All sequences used ECG gating with acquired slice thickness = 3-mm. The VFA-TSE sequence was acquired with a slab-selective excitation, 16 partitions, sampling bandwidth = 501 Hz/px, echo spacing = 2.74 ms, in-plane spatial resolution matched to the 3D uSSFP acquisition, with and without blood suppression of 50 or 100 mT/ms applied along all three coordinate axes. Both DIR-TSE and VFA-TSE were acquired with 2 signal averages.

### Qualitative image analysis

Images were reviewed by a single reader with more than 10 years of CMR experience. Image quality was scored subjectively according to a 4-point scale: (1) non-diagnostic, aortic wall not assessable due to artifacts; (2) fair, with moderate artifacts; (3) good, with mild artifacts; and (4) excellent image quality with negligible artifacts. For evaluation of VFA-TSE acquired with and without blood suppression, only the best quality image series was analyzed. Scores were analyzed using Friedman and post-hoc Wilcoxon signed-rank tests. P values less than 0.05 were considered statistically significant.

### Quantitative image analysis

Region-of-interest analysis was performed using an Intelerad PACS workstation (Montreal, Canada). Tissue signal and standard deviation were measured in the air anterior to the chest wall, in the subcutaneous fat, within the paraspinal muscles, within the lungs, and within the posterior wall and lumen of the mid-thoracic descending aorta. Since parallel imaging was used, the true signal-to-noise ratio (SNR) cannot be determined without additional measurements [17]. There, we refer to the apparent signal-to-noise ratio (aSNR), defined as tissue signal S/σ_air_ and the apparent contrast-to-noise ratio (aCNR) between two tissues, defined as (S_1_ – S_2_)/σ_air_.

## Results

### Bloch equation modeling

Figure 1C and D show the results of the numerical simulations. Simulations show that small flip angles (< 10°) provide little vessel wall-to-blood pool contrast irrespective of the gradient spoiler factor, whereas a flip angle of approximately 40° coupled with a spoiler factor of approximately 0.2 to 0.3 optimized vessel wall-to-blood pool contrast. Given that the specific absorption rate limited the maximum flip angle to about 35°, we used flip angles in the range of 22 to 33° for our study.

### 3D uSSFP of the heart and great vessels

For breath-hold 3D uSSFP, scan time was ~ 16 to 24 s depending on heart rate. Image quality was good-to-excellent in all subjects. The image quality score (mean ± SD) was 3.9 ± 0.4. aSNR measurements were as follows: aortic wall = 109 ± 65; aortic lumen = 14 ± 11; lung = 15 ± 11; fat = 122 ± 70; muscle = 90 ± 67. aCNR measurements were as follows: aortic wall-to-lumen 95 ± 56; aortic wall-to-lung = 95 ± 57; aortic wall-to-fat = − 13 ± 69; aortic wall-to-muscle = 19 ± 41; aortic lumen-to-lung =  −  0.6 ± 5.2; aortic lumen-to-fat = − 109 ± 64; aortic lumen-to-muscle = − 76 ± 57.

**Fig. 2:**
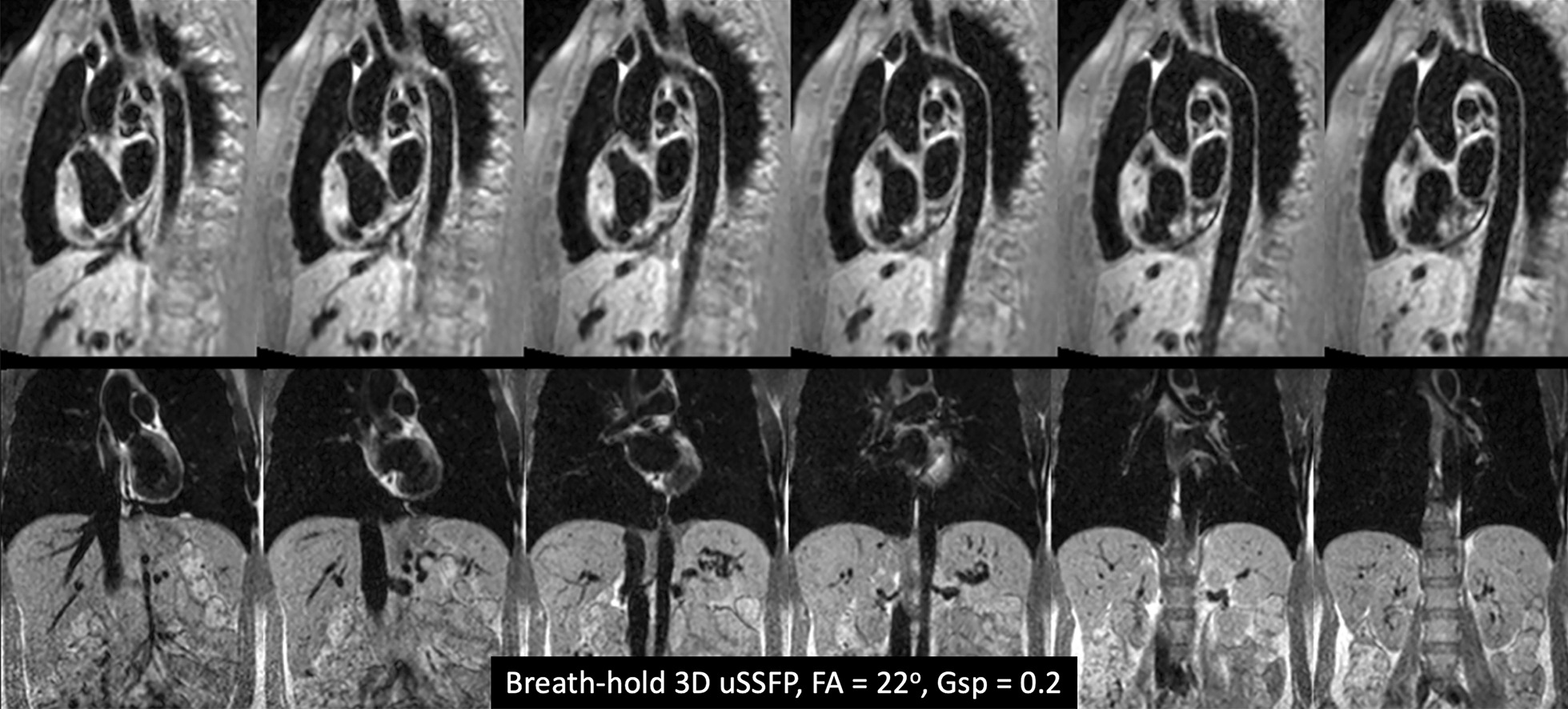
Healthy subject. Oblique sagittal (top) and coronal (bottom) breath-hold 3D uSSFP (6 images shown out of 24 acquired), acquired with flip angle (FA) = 22° and gradient spoiler factor (Gsp) = 0.2. There is uniform blood pool suppression in the chest and abdominal vessels without evidence of banding artifacts from off-resonance effects. The aortic wall is well demonstrated in the oblique sagittal views, while the upper abdominal vasculature is well shown in the coronal views

An example of breath-hold, ECG-gated 3D uSSFP of the great vessels in a healthy subject is given in Fig. 2. Within the chest, the blood pool signals in the heart, lungs, and great vessels appeared uniformly dark, as was the case for visualized portions of the abdominal vessels. No artifacts from off-resonance effects were observed.

**Fig. 3:**
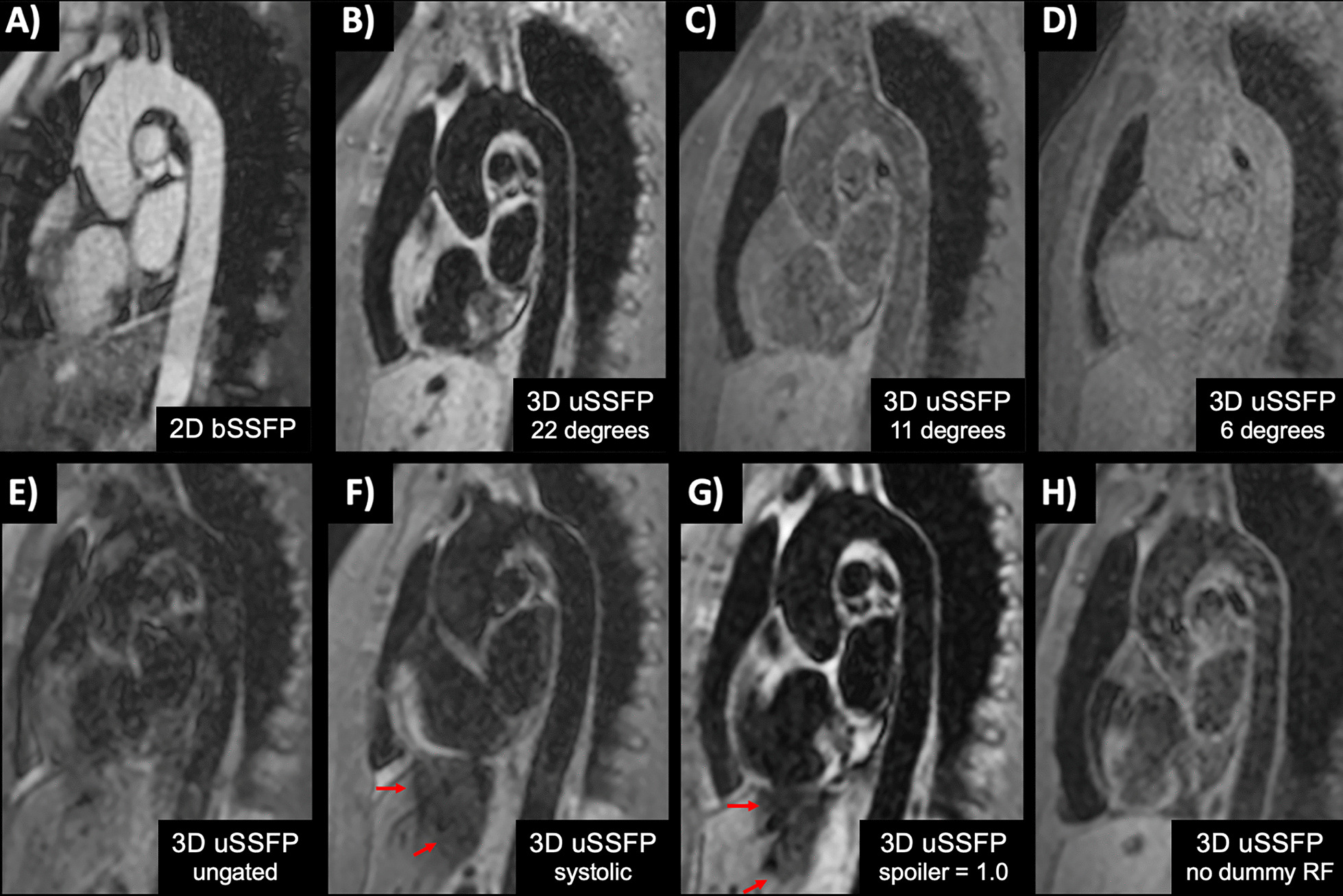
Pulse sequence comparisons in a healthy subject. **A** 2D bSSFP. **B** 3D uSSFP, flip angle = 22°. **C** 3D uSSFP, flip angle = 11°. **D** 3D uSSFP, flip angle = 6°. **E** 3D uSSFP, ungated. **F** 3D uSSFP, systolic gated. **G** 3D uSSFP, gradient spoiler factor = 1.0. **H** 3D uSSFP, no dummy radiofrequency (RF) pulses. Note that **B**–**F** were acquired with a gradient spoiler factor = 0.2 and 60 dummy RF pulses using a constant flip angle of 22°, the same flip angle as used for the RF excitation. With systolic gating or an increased gradient spoiler factor, signal loss due to transmitted cardiac pulsations is apparent in the left lobe of the liver (arrows)

The impact of variations in sequence parameters is shown in Fig. 3. At low flip angles, the images appeared proton density-weighted with gray blood contrast. As the flip angle increased, the blood pool signals decreased, appearing uniformly dark at a value of 22° (Fig. 3B–D). ECG gating had a substantial impact on image quality. With a diastolic-gated acquisition, the aortic wall was sharply delineated, as were blood vessels in the upper abdomen (Fig. 3B). By contrast, with a continuous ungated 3D uSSFP acquisition, there were extensive ghost artifacts with poor vascular detail (Fig. 3E). With a systolic-gated acquisition, there was increased blurring of the heart and aortic wall along with a loss of signal in the left lobe of the liver, presumably due to transmitted cardiac pulsations (Fig. 3F). Both flow spoiling and motion sensitivity increased with the moment of the gradient spoiler (Fig. 3G). A gradient spoiler factor of 0.2 provided a reasonable compromise between flow spoiling and excessive bulk motion sensitivity, so this value was used for patient studies. For ECG-gated acquisitions, dummy RF pulses were needed to obtain adequate blood pool suppression (Fig. 3B). Without them, the blood pool was not adequately suppressed (Fig. 3H).

**Fig. 4:**
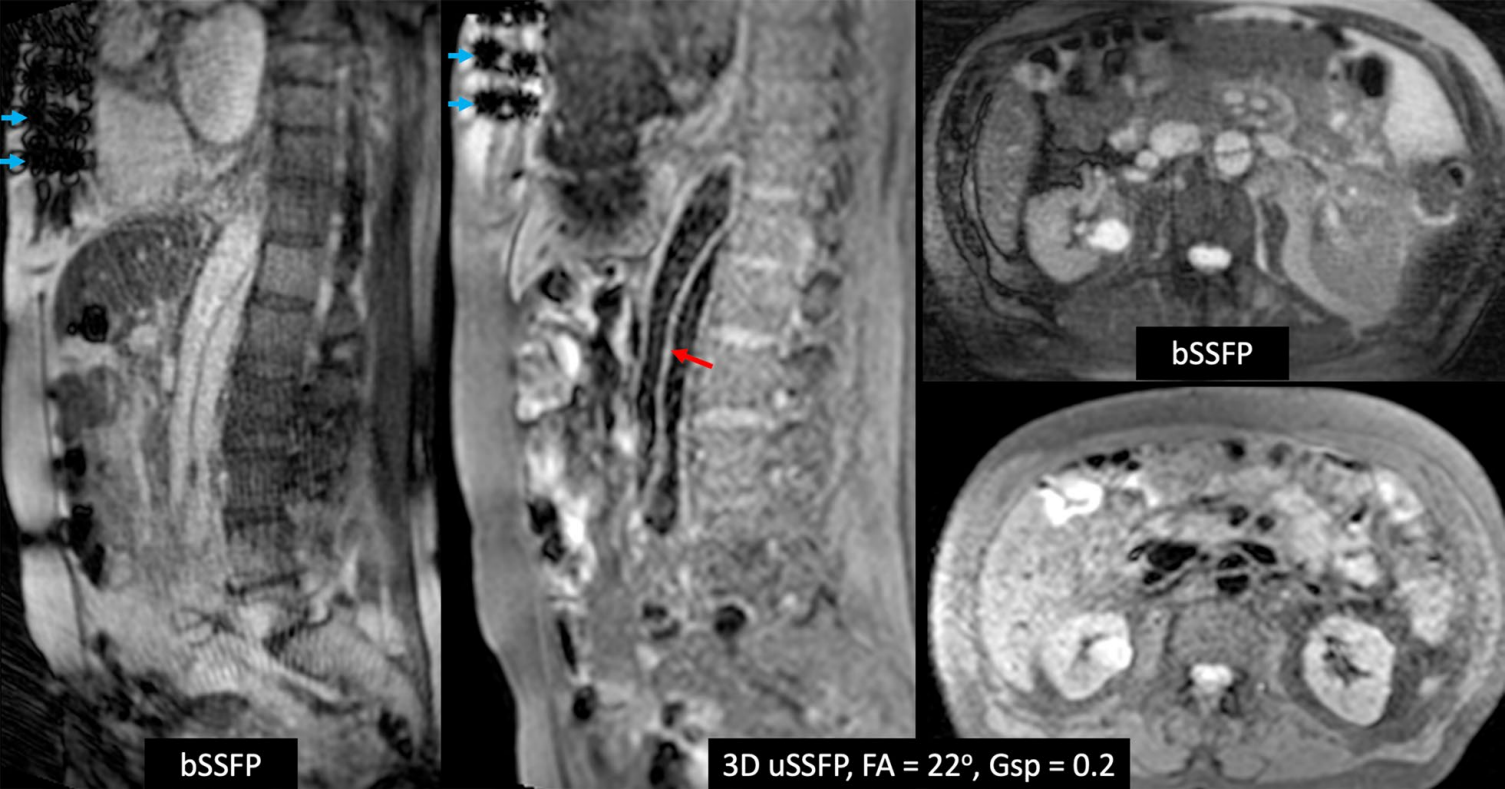
Patient with type B aortic dissection imaged using 2D bSSFP and 3D uSSFP (flip angle = 22° and gradient spoiler factor = 0.2). Both the false and true lumens along with the dissection flap (red arrow) are well demonstrated using breath-hold 3D uSSFP. Note that off-resonance artifacts (short blue arrows) from sternal wires are substantially reduced with 3D uSSFP compared with 2D bSSFP

**Fig. 5:**
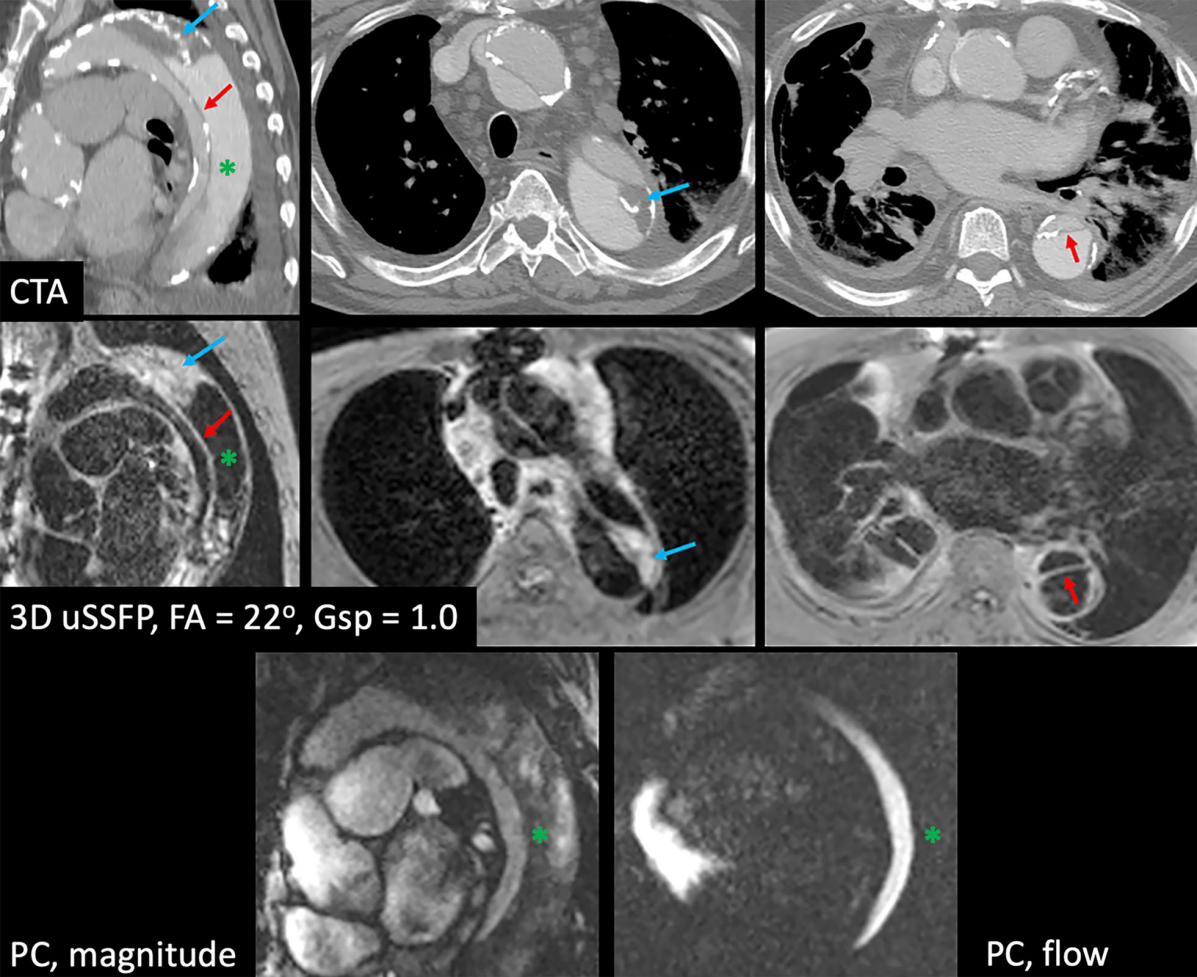
Repaired type A aortic dissection with very slow flow and thrombus (blue arrow) in the false lumen. *Top row* computed tomography angiography. *Middle row* 3D uSSFP. *Bottom row* Peak systolic magnitude (left) and flow (right) images from a phase contrast (PC) cine acquisition with in-plane velocity encoding sensitivity of 150 cm/s show negligible flow in the false lumen (*). Nonetheless, 3D uSSFP acquired with a gradient spoiler factor = 1.0 was able to suppress the blood pool signal in the false lumen, while the dissection flap (red arrow) is well demonstrated. Chronic intraluminal thrombus appears relatively bright with 3D uSSFP, as does mediastinal adenopathy

The blood pool signal in the thoracic aorta was uniformly suppressed using 3D uSSFP in all patients with normal caliber vessels, as well as two cases of aortic aneurysm and two cases of aortic dissection (Fig. 4). In two patients, the use of an increased gradient spoiler factor was helpful to improve blood pool suppression [0.5 in a patient with aortic aneurysm; 1.0 in a patient with an aortic dissection and very slow flow in the false lumen (Fig. 5)].

**Fig. 6:**
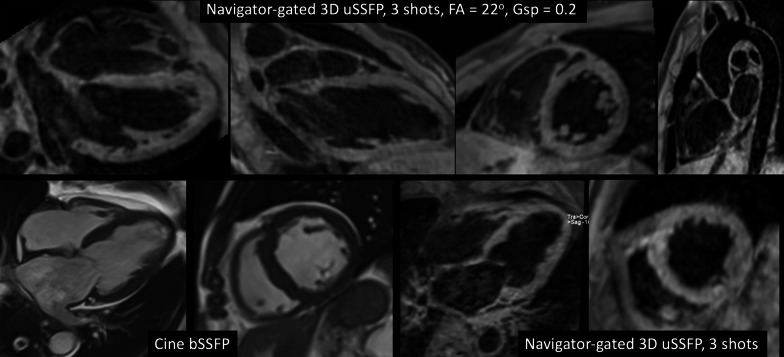
Examples of free-breathing, navigator-gated 3D uSSFP. *Top row* Healthy subject. RR interval was approximately 1 s. Scans were acquired in a sagittal orientation with multiplanar reconstructions performed in standard cardiac orientations (4-chamber, 2-chamber, short axis), as well as along the long axis of the aorta. There is uniform blood pool suppression without evidence of motion artifacts, allowing detailed evaluation of cardiac and aortic morphology. *Bottom row* Patient with dilated cardiomyopathy. RR interval was approximately 0.9 s. Diastolic phase cine bSSFP images in the 4-chamber and short axis orientations are compared with the corresponding double oblique multiplanar reconstructions from 3D uSSFP. Cardiac morphology is again well shown on the dark blood images without evidence of significant motion artifacts

For navigator-gated 3D uSSFP, scan time was 5 min 58 s in a healthy subject and 4 min 25 s in a patient. Excellent dark blood image quality was obtained, with little apparent loss of image quality when multiplanar reformations were reconstructed along standard cardiac planes (Fig. 6).

### Comparison of breath-hold dark blood imaging techniques

**Fig. 7:**
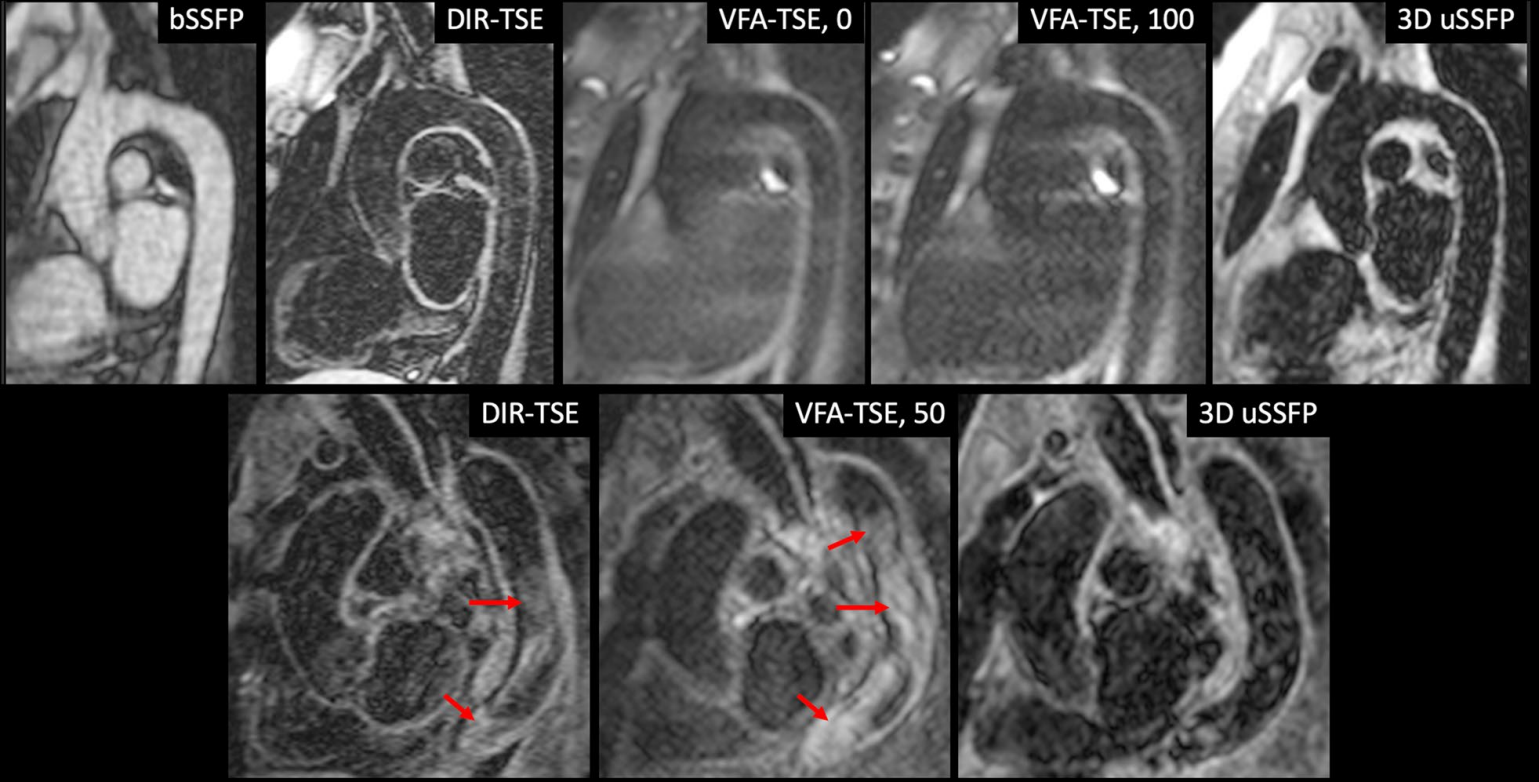
Comparison of breath-hold black blood imaging techniques in two patients undergoing CMR. Top row: 44-year-old patient with normal caliber thoracic aorta and left ventricular ejection fraction of 62%. From left to right: 2D bSSFP; dual inversion-turbo spin echo (DIR-TSE); variable flip angle (VFA)-TSE without blood suppression; VFA-TSE with blood suppression of 100 mT/ms applied along all three coordinate axes; 3D uSSFP. The blood pool signal in the thoracic aorta is well suppressed with all techniques. The wall of the descending aorta is sharply delineated with 3D uSSFP but appears blurred with both VFA-TSE acquisitions. The wall is well shown by DIR-TSE in the upper and middle segments but is less distinct in the lower segment. Moreover, the ascending aorta is less well depicted with VFA-TSE than by the other dark blood techniques. Bottom row: 86-year-old patient with aneurysmal thoracic aorta and severely decreased left ventricular ejection fraction of 26%. From left to right: DIR-TSE; VFA-TSE with blood suppression of 50 mT/ms applied along all three coordinate axes; 3D uSSFP. The blood pool is well suppressed with 3D uSSFP, but substantial intraluminal signal (arrows) remains with DIR-TSE and VFA-TSE

The image quality scores (mean ± SD) for 3D uSSFP, DIR-TSE and VFA-TSE were, respectively: 3.9 ± 0.4, 3.1 ± 0.9, and 2.6 ± 0.9 (P = 0.013, Friedman test). A statistically significant difference was found between uSSFP and VFA-TSE (P = 0.015) whereas a trend was found between uSSFP and DIR-TSE (P = 0.059). Typical image quality is illustrated in the top row of Fig. 7. While all three techniques were usually effective at suppressing the blood pool signal within the thoracic aorta, the aortic wall appeared blurred using VFA-TSE. In one patient with an aneurysmal thoracic aorta and low ejection fraction, the blood pool appeared uniformly dark with 3D uSSFP but appeared inhomogeneous with VFA-TSE despite the application of blood suppression (Fig. 7, bottom row). The blood pool in this patient also appeared inhomogeneous with DIR-TSE.

### 3D uSSFP of the lung and mediastinum

**Fig. 8:**
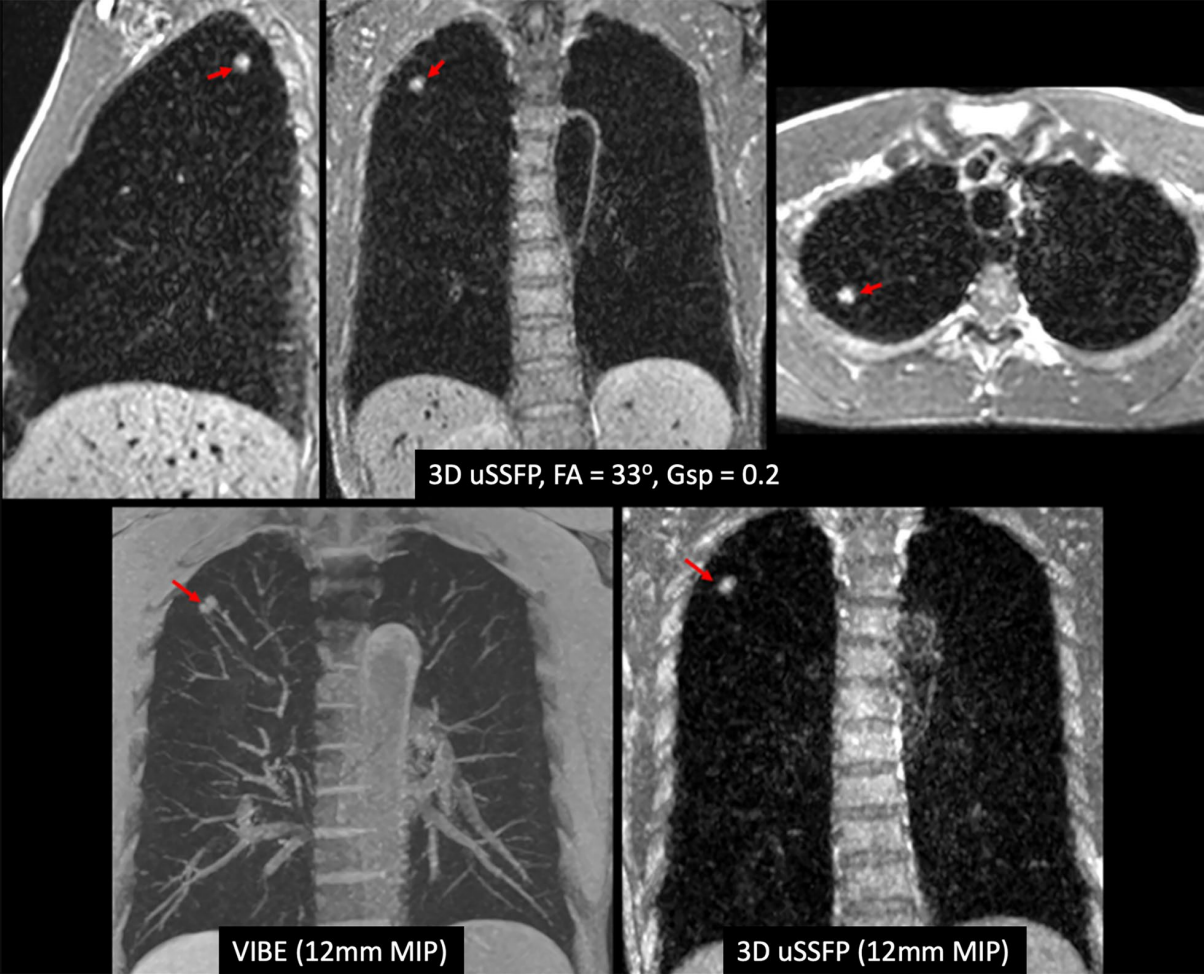
Example of dark blood imaging of the lungs using non-contrast, breath-hold 3D uSSFP. *Top row* 7-mm solid lung nodule is well demonstrated by 3D uSSFP acquired in three orthogonal scan planes. Note that the blood vessels appear uniformly dark. *Bottom row* Comparison of 12-mm thick maximum intensity projections using VIBE (left) and 3D uSSFP (right). While the lung nodule can be seen with both techniques, the lesion is much more conspicuous with 3D uSSFP due to the low blood pool signals and resultant sparsity of the images

**Fig. 9:**
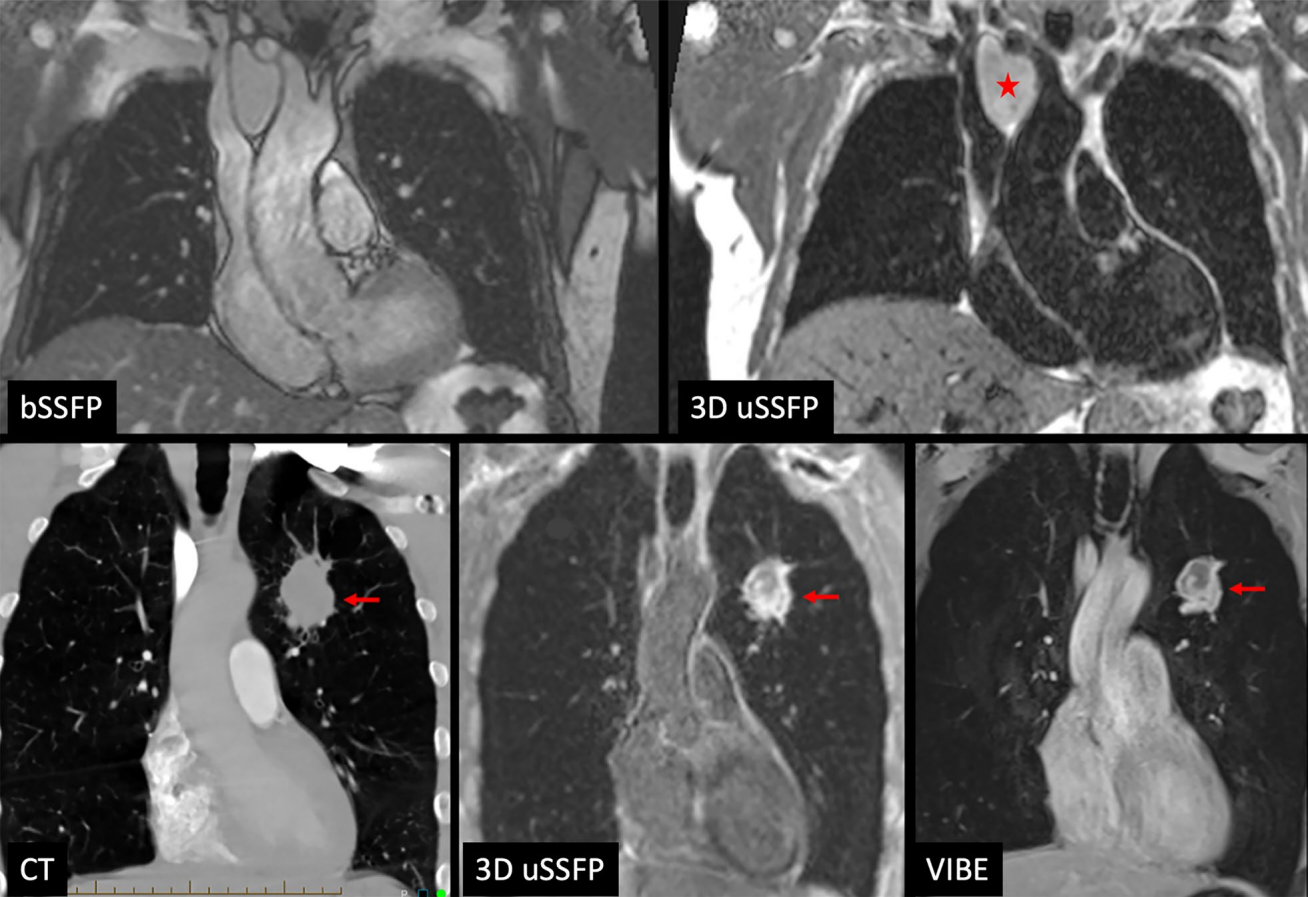
Breath-hold 3D uSSFP in two different patients. *Top row* Non-contrast images in a patient with a benign mediastinal cyst (*). Left: 2D bSSFP; right 3D uSSFP. In the 2D bSSFP image, the cyst is difficult to distinguish from the adjacent, nearly isointense brachiocephalic vein, aortic arch, and branch vessels, but is easily visualized with 3D uSSFP due to the low blood pool signals. *Bottom row* Postcontrast images in a patient with a necrotic, biopsy-proven non-small cell lung cancer (red arrows). Left: CT; middle: 3D uSSFP; right: VIBE. Since the 3D uSSFP images were obtained after contrast administration, the blood pool appears gray rather than black. Nonetheless, 3D uSSFP better demonstrates the tumor spiculations and provides higher contrast between the tumor and cardiovascular structures than VIBE

Breath-hold 3D uSSFP was performed in three patients with lung lesions and one patient with a mediastinal lesion. Lung and mediastinal lesions appeared highly conspicuous compared with the low blood pool signals within the heart and blood vessels. The lesions were less conspicuous when imaged with a bSSFP or volumetric interpolated breath-hold examination (VIBE) pulse sequence due to the brighter blood pool signals (Fig. 8). In two patients where 3D uSSFP was acquired after contrast administration (1 subject with lung cancer, 1 subject with pulmonary sarcoid), the blood pools appeared gray, but still provided adequate lesion-to-vessel contrast (Fig. 9).

## Discussion

Our initial results suggest that breath-hold, ECG-gated 3D uSSFP consistently provides good-to-excellent image quality for dark blood evaluation of the great vessels of the chest, while a free-breathing, navigator-gated implementation offers the potential for dark blood morphological evaluation of the heart. The aortic wall was well demonstrated in all subjects. In the setting of very slow blood flow, the use of an increased gradient spoiler factor could be helpful to obtain uniform blood pool suppression and was not observed to have any deleterious effect on the conspicuity of the aortic wall. Moreover, 3D uSSFP was insensitive to off-resonance effects (unlike bSSFP) so that no banding artifacts were observed [18].

3D uSSFP proved advantageous compared with two established dark blood imaging techniques (2D DIR-TSE and 3D VFA-TSE). For instance, compared with VFA-TSE, 3D uSSFP showed improved sharpness of the aortic wall. It was also more efficient, permitting the acquisition of 50% more slices in each breath-hold since VFA-TSE requires the use of signal averaging to reduce free induction decay artifacts. Comparing 3D uSSFP with DIR-TSE, aortic wall sharpness was similar, but DIR-TSE only permitted a single slice to be acquired in each breath-hold versus 24 slices with 3D uSSFP.

bSSFP is intrinsically motion-compensated so that flowing spins appear bright, while uSSFP dephases flowing spins so that they appear dark [19, 20]. Whereas bSSFP is routinely used for the evaluation of the cardiovascular system [21, 22], uSSFP has few if any clinical applications aside from the niche application of MR fingerprinting [23]. The main reason is that legacy implementations of uSSFP tend to be excessively motion sensitive and yield poor image quality. In fact, a literature search for dark blood vascular imaging using uSSFP revealed only a single report which dates back 27 years [24].

The 3D uSSFP technique used here was derived from the uT_1_RESS sequence that we recently described for contrast-enhanced imaging of brain tumors [14]. Both techniques use a high sampling bandwidth, short echo spacing, very short echo time, weak dephasing gradient, and a single-shot acquisition along the phase-encoding direction. However, 3D uSSFP diverges from uT_1_RESS in several key aspects, including: (i) use of ECG gating to synchronize the data acquisition to the diastolic phase of the cardiac cycle; (ii) application of a series of constant flip angle dummy pulses to drive the spins into a steady-state prior to the acquisition of each shot; (iii) elimination of the contrast-modifying partial saturation RF pulse that was used in the brain to suppress the signal from long T2 species, specifically cerebrospinal fluid and edema; (iv) use of a slab-selective rather than spatially non-selective RF excitation, along with a reduced flip angle; and (v) acquisition of a smaller number of 3D partitions to reduce the scan duration to a comfortable breath-hold.

The capability for a short breath-hold scan makes it convenient to incorporate 3D uSSFP into a routine CMR protocol, as was done in the present study. By comparison, two previous reports using VFA-TSE to image the aortic wall [13, 25] reported the use of free-breathing acquisitions with scan times > 7 min. While scan times for 3-shot navigator-gated 3D uSSFP are also relatively lengthy, the superior temporal and spatial resolution enabled detailed depiction of whole-heart morphology in source images and double oblique multiplanar reformations, which was not possible using the single-shot breath-hold approach.

We found that ECG gating was essential to obtain satisfactory image quality with 3D uSSFP. The use of ECG gating avoids vessel wall displacement during the systolic pulse wave as well as ghost artifacts from accelerating intraluminal spins and promotes consistent signal across uSSFP partitions. Previous reports using other imaging techniques demonstrated that ECG gating improved image quality scores along with the clarity of the aortic wall and mediastinum [18, 26].

The primary goals of this initial pilot study were to determine pulse sequence parameters and demonstrate the feasibility of applying 3D uSSFP in the chest. Further study will be required to assess the clinical utility of this novel imaging method. For instance, breath-hold 3D uSSFP might prove useful during CMR exams to better assess incidental lung and mediastinal lesions that are often poorly demonstrated using standard-of-care techniques, or to measure aortic plaque volumes. The navigator-gated version could be used as an adjunct to bright blood cine imaging for the evaluation of cardiac morphology in patients with hypertrophic cardiomyopathy, arrhythmogenic right ventricular cardiomyopathy, or other cardiac disorders. As with bSSFP [27], a T2-weighted preparation module can be applied to highlight edema and other pathology, while the addition of a saturation or inversion preparation may permit dark blood evaluation of late gadolinium enhancement. 3D uSSFP could have useful applications in other vascular systems as well. Like previously described dark blood imaging techniques [10], it could potentially be used to detect and characterize mural plaque and thrombus in the carotid arteries and intracranial circulation, or to detect arterial wall inflammation in vasculitis or vulnerable plaque.

### Limitations

This feasibility study was limited by several factors, including: (1) the patient cohort was small and heterogeneous. (2) Only a single reader performed qualitative analysis, whereas two or more readers would be preferred. (3) We measured the aSNR and aCNR, which may not accurately reflect the true SNR and CNR because of the use of parallel imaging. (4) We only applied parallel acceleration along a single dimension. Further image quality improvements and increased spatial coverage could be obtained by using 2D parallel acceleration [28] or compressed sensing [11, 29]. (5) The two patients in whom navigator-gated 3D uSSFP of the heart was obtained had relatively slow heart rates. The technique will likely show more motion artifacts in patients with rapid heart rates in whom there might not be a sufficiently long quiescent period within the cardiac cycle. (6) The current implementation of 3D uSSFP uses a Cartesian 3D k-space trajectory. However, a stack-of-stars k-space trajectory might be advantageous to reduce the sensitivity to respiratory motion and eliminate ghost artifacts [30, 31], so long as care is taken to avoid having the radial trajectory disrupt the steady-state magnetization [32]. (7) We have limited experience using the VFA-TSE technique in the chest. It is likely that image quality could be improved through further optimization of the sequence parameters.

## Conclusion

In this pilot study, we have demonstrated the feasibility of using ECG-gated 3D uSSFP for dark blood imaging of the heart, great vessels, and lungs. Further study will be required to fully optimize the technique and to assess clinical utility.

## Data Availability

The simulation code used for Bloch equation modeling can be provided upon request. Ethics approval and consent to participate The study was approved by the hospital institutional review board (IRB). Written informed consent was obtained for volunteers (n = 5). Waiver of consent was obtained for patients (n = 26) undergoing a clinically indicated cardiac MRI exam during which additional dark blood sequences were obtained.

## Abbreviations

3D: Three-dimensional
aCNR: Apparent contrast-to-noise ratio
aSNR: Apparent signal-to-noise ratio
bSSFP: Balanced steady-state free precession
CMR: Cardiovascular magnetic resonance
DIR: Dual inversion
ECG: Electrocardiogram
RF: Radiofrequency
TSE: Turbo spin echo
uSSFP: Unbalanced steady-state free precession
uT_1_RESS: Unbalanced T1 Relaxation-Enhanced Steady-State
SNR: Signal-to-noise ratio
CNR: Contrast-to-noise ratio
